# Attitudes, Practices and Understanding of health workers and caregivers regarding the relationship between severe pneumonia and malnutrition in children: A Qualitative Study

**DOI:** 10.21203/rs.3.rs-3386868/v1

**Published:** 2023-10-09

**Authors:** Damalie Nalwanga, Robert Opika Opoka, Andrew Sentoogo Ssemata, Lawrence Kakooza, Andrew Kiggwe, Victor Musiime, Sarah Kiguli

**Affiliations:** Department of Paediatrics and Child Health, School of Medicine, College of Health Sciences, Makerere University; Department of Paediatrics and Child Health, School of Medicine, College of Health Sciences, Makerere University; MRC/UVRI & LSHTM Uganda Research Unit; Makerere University Lung Institute; Makerere University Lung Institute; Department of Paediatrics and Child Health, School of Medicine, College of Health Sciences, Makerere University; Department of Paediatrics and Child Health, School of Medicine, College of Health Sciences, Makerere University

**Keywords:** Pneumonia, Malnutrition, Caregivers, Health workers, perceptions, Nutrition, practices

## Abstract

**Background:**

Severe Pneumonia is still the leading cause of morbidity and mortality among children worldwide. Many children with severe pneumonia are reported to die in hospital as well as following discharge due to malnutrition. Severe pneumonia is a catabolic illness, which predisposes to severe malnutrition. WHO and United Nations Children’s Fund (UNICEF), recommend ‘continued’ feeding but do not give any specific recommendations for nutritional support. This could influence health workers’ and caregivers’ attitudes, practices and understanding regarding the topic. This study aimed to explore the attitudes, practices and understanding of health workers regarding the relationship between severe pneumonia and malnutrition.

**Methods:**

We conducted an exploratory qualitative study among health workers and caregivers of children hospitalized with severe pneumonia at Mulago National Referral Hospital in Uganda. Data were collected using focus-groups involving caregivers and key informant interviews with health workers and analysed using the content-thematic analysis approach. Both manual coding and Atlas Ti software were used to support the analysis.

**Results:**

Some of the health workers and caregivers were aware of the relationship between severe pneumonia and malnutrition to various degrees, citing reduced appetite, difficulty in breathing and persistent vomiting as pathways to malnutrition in patients with severe pneumonia, which called for a balanced diet and more frequent breastfeeding. Suppressed immunity in malnourished children was mentioned as the pathway to severe pneumonia. Some caregivers confessed not knowing anything about the relationship between the two conditions.

**Conclusion:**

Attitudes, practices and understanding regarding the deadly relationship between severe pneumonia and malnutrition among care givers could further be improved by health education and mass sensitization. Clarifying practice guidelines could further enhance attitudes and practices of health workers to reduce preventable pneumonia deaths.

## Background

Severe Pneumonia is the leading cause of morbidity and mortality among children worldwide. In Africa, the incidence of severe pneumonia is estimated at one episode per three child years with a case fatality of 9.8% (1). In 2013 Liu et al estimated that 1 in 150 children under five years died of severe pneumonia (2). Pneumonia is estimated to account for 14% of all-cause mortality in children under 5 years of age (3). Children with severe acute malnutrition (SAM) have a higher risk of dying (2, 4). In addition to the concerning statistics on inpatient mortality from severe pneumonia, many children are reported to die even following discharge (5). A study by Ngari et al. conducted in Kenya among hospitalized children found that 364 (8.7%) children died in hospital and following discharge. Post discharge mortality was higher among children previously admitted for severe pneumonia compared to other diagnoses with a hazard ratio of 2.5 (95% Cl 1.2, 5.3), and majority (52%) were attributed to malnutrition (6). Increased mortality from respiratory illnesses in linked to lower mid-upper arm circumference (6, 7).

**Figure 1:**
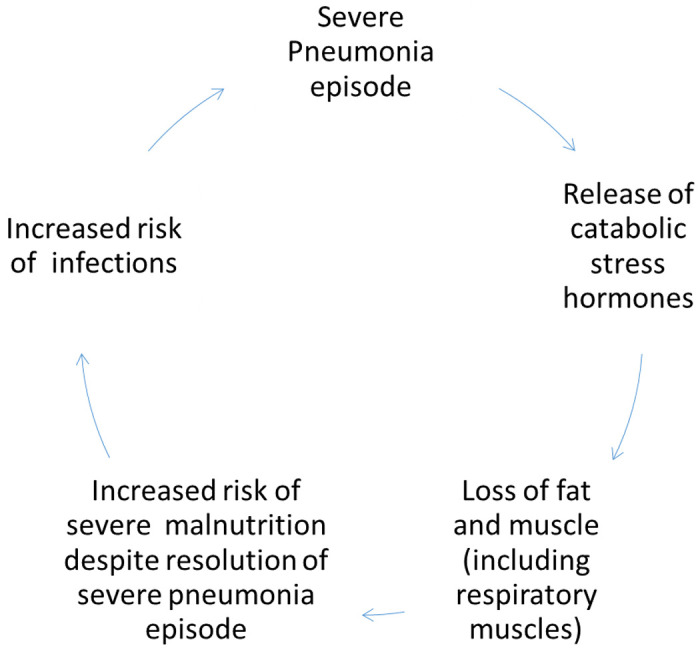
Cyclical relationship between severe pneumonia and severe malnutrition

Severe Pneumonia is a catabolic illness, resulting from the release of stress hormones to increase energy production and support the work of breathing. Body stores are utilized for metabolism resulting in rapid skeletal muscle breakdown and cachexia or ‘wasting’ (8). Furthermore, children appetite and therefore reduced intake during the pneumonia episode and in the recovery period. These processes therefore predispose children to poor nutritional status and may lead severe malnutrition through loss of body fat and muscle, which in turn increases their risk of acquiring severe infections like severe pneumonia, and death (a vicious cycle as summarized in Fig. 1 below).

The World Health Organization (WHO) and United Nations Children’s Fund (UNICEF), under the ‘Treat’ element of Protect, Prevent and Treat framework for severe pneumonia treatment recommend ‘continued’ feeding to try to mitigate the risks of severe acute malnutrition in children with severe pneumonia (9). However, they do not give any specific recommendations or guidance on nutritional support for children hospitalized for severe pneumonia. The lack of clear guidance could influence health workers’ attitudes and practices concerning the relationship between pneumonia and malnutrition when caring for children with severe pneumonia.

As part of the integrated management of childhood illnesses (IMCI), health workers offer general feeding advice to children’s caregivers. However, majority of the health workers in Uganda do not assess current feeding before giving advice to the caregivers of children with severe illness like severe pneumonia (10). This could reveal a lack of understanding of the relationship between severe illness and severe malnutrition influencing their attitudes and practice. Even though caregivers do not always practice everything advised by health workers, their attitudes and practices are shaped in some ways by the health workers’ advice.

Understanding caregivers’ and health workers, attitudes, and practices towards the complex relationship between severe pneumonia and malnutrition is crucial for designing educational materials to interrupt the deadly vicious cycle between pneumonia and malnutrition. This study aimed to assess the attitudes and practices of health workers and caregivers about the relationship between pneumonia and malnutrition.

## Methods

### Study design.

We conducted an exploratory qualitative study using focus group discussions (FGDs) with caregivers of the children and key informant interviews (KIIs) with the health workers.

#### Study setting

The study was conducted at Mulago National Referral Hospital in Kampala, Uganda. It is the largest public hospital in Uganda and serves as a teaching facility for Makerere University College of Health Sciences. The hospital is located on Mulago hill in the northern part of the city of Kampala, immediately west of the Makerere University College of Health Sciences. It is approximately five kilometres, by road, north–east of Kampala’s central business district. The hospital receives the most critically ill patients from all over the country and provides primary care to nearby communities. Children with severe pneumonia are admitted at the emergency ward (Acute Care Unit) before being transferred to the cardiorespiratory ward (Ward 16C), and are cared for by nurses, medical officers, senior house officers (residents in paediatrics speciality training) and paediatricians.

#### Study participants

Health workers and caregivers of children hospitalized for severe pneumonia who could speak English and/or Luganda were consented for the study. Health workers are health workers who are directly involved in paediatric patient care e.g., nurses, medical officers, senior house officers and paediatricians. Caregivers were considered as parents or guardians involved in the day-to-day care of children hospitalized for severe pneumonia. The selection criteria are summarized in **supplementary table 1**.

#### Data collection

Qualitative data was collected from both health workers, i.e., medical officers, nurses, senior house officers, paediatricians and caregivers of children hospitalized for severe pneumonia using key informant interviews (KIIs) and focus-group discussions (FGDs) respectively. To ensure that varying views were explored, we conducted six FGDs with 6–8 purposively selected caregivers of children hospitalized for severe pneumonia with different ages and gender constituting each FGD. We also conducted 14 KIIs with the health workers involved in the management of children hospitalized for severe pneumonia. We planned to continue recruiting participants, but this was not necessary as saturation was reached. We used a semi-structured guide with a pre-tested list of open-ended questions, arranged in a logical sequence for all FGDs and KIIs. The study team established rapport with study participants before commencing each FGD and KII, conducting it in English and/or Luganda in the presence of an experienced moderator. FDGs and KIIs were audio-recorded using a digital recorder and a separate note taker was present. Each FGD and interview lasted 60–80 minutes. KIIs and FDGs were conducted until a point of saturation where no new information was generated by further data collection for both groups. All study procedures were conducted under strict COVID-19 precautions including use of masks, social distancing, and hand hygiene.

#### Data Analysis

Audio recordings from the KIIs and FGDs were transcribed verbatim by the study staff. All FGDs and KIIs were transcribed in English and those conducted in Luganda were translated. The transcripts were read alongside the note taker’s field notes, while making brief notes at the points of interest and relevant information captured. The transcripts and audio recordings were reviewed to ensure accuracy. Data were analysed using the content-thematic analysis approach (11). Both manual coding and Atlas Ti software was used to support the analysis (12). Preliminary data analysis was conducted as the data collection process is ongoing. Upon initial coding of the transcripts, a codebook was developed and refined during data collection as recommended by Braun et.al (21). The codebook was used to deduct information from the transcripts to answer the proposed research questions. All disagreements were discussed, and a consensus was reached on the overall analysis.

#### Ethical considerations

The study was approved by the Mulago Research Ethics Committee (MREC 1933) and Uganda National Council for Science and Technology (UNCST: SS803ES). Prior to data collection, respondents’ verbal and written consent were sought. They were informed about the study procedures and made to understand that participation was voluntary and refusal to participate would not attract any penalty or adversely impact their work. To ensure confidentiality, we did not use personal identifiers.

## Results

In summary, we found that caregivers and health workers had differing attitudes and practices and understanding related to malnutrition and severe pneumonia.

### Caregivers’ understanding, attitudes, and practices.

The caregivers’ understanding, attitudes, and practices regarding the relationship between severe pneumonia and malnutrition in children varied widely, as detailed below.

### Pneumonia and malnutrition are interconnected.

Many of the caregivers reported that a malnourished child can easily develop pneumonia. They mentioned that if a child is not feeding well or is malnourished, their immunity will be compromised; when they catch a cough, it will escalate to weight loss, difficulty in breathing, constant headaches, and other symptoms, and eventually, the child will get pneumonia.

“I hardly believe the child can normalize after going through all that even after receiving medication. Because the child takes a lot of time when is not eating and that means in just one week he can lose the weight because of the cough, difficulties in breathing, sneezing all the time, headache so I think if the child becomes malnourished it is easy for him to get pneumonia”. (Mother)

Some caregivers believed that pneumonia leads to malnutrition, citing those symptoms of pneumonia, such as loss of appetite and vomiting, which reduce food intake and absorption, lead to malnutrition if not addressed immediately.

“I noticed he was not eating anything and vomiting as you know that pneumonia comes with a lot of fever and loss of appetite. When the baby was not eating, I observed the change in physical appearance and drop in body weight and that is when I knew that something was wrong. By the time I came into the hospital, he was already malnourished.” (Mother)

From the caregivers’ perspective, the possibility of pneumonia leading to malnutrition was attributed to the inability of the sick child to consume the required food nutrients due to lack of appetite and suffocation resulting from the pneumonia.

“… if a child does not feed well, he doesn’t want whatever you give, how will they get the energy? The pneumonia will lead to malnutrition. When the mother puts him on the breast to suckle, they cannot because they are not breathing well because of that illness [pneumonia]. He wants to suckle the breast, but the illness prohibits him and that makes him malnourished.” (Father)

The caregivers felt that when a child suffers from pneumonia and their food intake decreases, their body weight will drop, and they will eventually become malnourished. For other caregivers, pneumonia at times presents with frequent vomiting, which increases the risk of becoming malnourished. As a result of this relationship, caregivers are careful about feeding their malnourished children.

“It also depends on how the parent takes care of the child because if the child is given body building food, the child cannot get malnourished even if he/she eats a little on the food. The child can have appetite, but you give him non-body building foods it can cause a child to get malnourished.” (Grandparent)

“I would see them being given mashed silver fish or groundnuts they are cooked and fed to them, also milk that’s what I used to see them being given - food that can take care of them well” (Father)

The caregivers believed that feeding the children certain foods would protect them from disease but cited that having the finances to buy the food is challenging.

“To avoid pneumonia and malnutrition calls for us to avoid the luxurious life and go back to our normal African food. For example, there are three types of foods you must serve in a meal, body building food, energy giving food and protection food all in one plate. Many fruits are needed because they protect the body from other diseases; however, they are expensive.” (Mother)

Other caregivers opt to breastfeed their babies exclusively because breast milk is believed to have sufficient nutrients to support child growth and prevent ill health. However, they cited the challenge of difficulties in breathing, making breastfeeding difficult for children with pneumonia and necessitating an increase in breastfeeding frequency.

“Preventing children from becoming malnourished, I breast feed him. He breastfeeds well, but I have not started him on solids because he is still young. I think when a child is young, you must breastfeed him up to 2 years of age. I breast feed him, but when he is breastfeeding, you see that he is breathing heavily, and that thing stops him from breathing well” (pg. 3 line 87-9089)

### Pneumonia and malnutrition are not linked.

Contrary to the above, some caregivers do not believe that malnutrition and pneumonia are in any way linked. Despite pneumonia being a leading cause of death in children, some caregivers are naïve/ignorant about pneumonia. They sometimes hear about the disease for the first time when their children are diagnosed with it.

“I don’t have much experience since it is the first time, they are telling me that he has pneumonia in the hospital… Most people in the community have no knowledge about pneumonia because even me at first, I couldn’t tell that it was pneumonia disturbing my child… may be we don’t believe that pneumonia can lead to malnutrition simply because of ignorance because when you lack knowledge you will not know how to deal with it.” (Mother)

Some caregivers mentioned that it is difficult to prevent pneumonia in children since some of the causes are not clearly known. While other caregivers took pneumonia so lightly and ignored its effect among children. As a result, they did not seek medical care.

“Where I live pneumonia is taken so lightly and not a disease. Many say you can’t prevent this condition as it is inherited from the parents. So many mothers in our village even when their children fall sick, they do not take them for treatment they suffer find ways of managing the sickness from home.”

(Grandparent)

Many caregivers usually fail to seek the right medical management for their children, resorting to alternative medicines and self-medication when the condition is still manageable and presenting to the hospital only if there is no improvement.

“Most caregivers believe in seeking care from the clinics or traditional healers than coming here to the hospital where there’s proper management. So that places the children at risk of having severe pneumonia and becoming malnourished.” (Father)

Some of the caregivers attributed the child’s illness to other causes based on the traditional beliefs held in the community. This delays the urgent management and treatment of children with severe pneumonia or malnutrition.

“In some villages, people who have their traditional norms, even when a child is visibly malnourished, will relate it to some cultural or traditional beliefs. Even when a child has a pneumonia, a terrible fever and is coughing, to them it was something traditional causing the malnutrition and the pneumonia.” (Mother)

“Actually, when you visit these people, they will tell you that it is not malnutrition or pneumonia. They say the disease is called “kidugavu,” [a condition attributed to witchcraft] and it is not an illness, so they believe that it is a demonic thing, so they believe that you must treat it locally. Therefore, when you go to the community to assess you are going to find very many children who have pneumonia, and they are malnourished, and they have never been brought to hospital.” (Father)

### Caregivers’ failure to seek the right treatment and management.

Health workers noted that many caregivers usually fail to seek the right medical management for their children. Some resort to alternative medicines and self-medication when the condition is still manageable thereafter seek medical advice once they feel there is no improvement at which stage the condition has escalated and is out of control.

“Most caregivers believe in seeking care from the dispensaries or traditional healers than coming here to the hospital where there’s proper management. So that places the children at risk of having severe pneumonia and becoming malnourished.” (Medical officer)

One of the key informants stated that many caregivers relate the child’s illness to other causes based on the traditional beliefs held in the community. It was noted that in some African traditional societies, disease is attributed to someone not wishing you well and relating the child’s illness to being bewitched. This delays the urgent management and treatment of the child with severe pneumonia or malnutrition.

“I had a parent here a few weeks ago, the child had severe pneumonia but to her it was a witchcraft spell cast on the child that was causing the malnutrition and the pneumonia.”(Nurse)

### Health workers’ understanding, attitudes, and practices.

The health workers expressed their understanding, attitudes and practices about severe pneumonia and malnutrition, as summarized below.

### Pneumonia and malnutrition are interrelated; one leads to another.

Many of the health workers mentioned that there is a significant association between pneumonia and malnutrition. They believe that these two conditions are closely linked to one another, as most of the children they received with the condition of pneumonia also had malnutrition.

“I would say that there’s a relationship because we get children who are both pneumonic and malnourished” (Medical officer)

“When Pneumonia is not treated, sometimes mothers at home when they see that the baby has flu or cough, they see it as a simple cough and the child coughs for more than two weeks and they still treat with other alternative medicines at home. Therefore, the child will lose the appetite and loses weight, and by the time they come to the hospital, they are already malnourished.” (Nurse)

They added that pneumonia affects nutrition by reducing intake in children while increasing their metabolism. This relationship makes it imperative that health workers encourage mothers/caregivers of children with pneumonia to feed their children adequately.

“If a child has pneumonia, they will have increased metabolism because they are using a lot of energy to breathe, they have fever, all of which take up the energy and the glucose from their bodies. Therefore, they need more than a normal child so they should be feeding more to keep up and if they don’t then they get malnourished. (Paediatrician)

“You are using the little food you have eaten because you must keep up with metabolism, you must keep up with the energy that is required for you to keep breathing… some babies when they are sick, they tend to take in very little because of loss of appetite and the baby tend not be very active to feed very well. So that is another way they can get malnutrition with severe pneumonia” (Medical officer)

“Of course, it is hard to tell what others think, but I know they also know that pneumonia can affect the nutrition of the child. This is because you will see them taking the initiative to advise these mothers on how to feed their children by taking enough fruit, water or even questioning the mothers if they have given the child some food or even breastfeeding the babies.” (Nurse)

The health workers further mentioned that recurrent pneumonia episodes increase the risk of malnutrition. This simply means that their intake is reduced/affected so often, and therefore, the child cannot recuperate.

“Most of my colleagues still know that pneumonia affects nutrition, that is why when a child comes in with pneumonia, we labour to attend to it, to ensure that the child’s intake is adequate, to prevent the child from getting malnourished. Yeah, so the wards I work with basically they know that pneumonia increases the risk of getting malnutrition more so if a child keeps on getting recurrent pneumonia” (Senior Housing officer)

“Those with recurring pneumonia usually have difficulty breathing, so their intake of food is also reduced. However, also with recurring infection, we all know that the body demands a lot and if not provided, it also leads to malnutrition” (Paediatrician)

According to some health care workers, malnutrition is accelerated by children having pneumonia and delaying their presentation to health facilities for appropriate management. One health care worker noted that even when caregivers realized that their child has had cough for a long time, for example, more than 2 weeks with no appetite and the child has lost weight, they would consider it ordinary cough.

“Most times this is not usual pneumonia where you come, I treat you with antibiotics and you go, n However, pneumonia that leads to malnutrition, are these children who have recurring symptoms for example they may be having recurring pneumonia that every other time they are in hospital and diagnosed with it.”(Senior Housing Officer)

Some believed that recurrent pneumonia leading to recurrent hospitalizations increases the risk of malnutrition, as healthy diets for children may be absent in the hospital.

“Most times this is not the usual pneumonias that we know where you come, I give you antibiotics and you go, no. However, the pneumonia that leads to malnutrition, are these children who have recurring symptoms for example they may be having a recurring pneumonia that every other time they are in hospital and diagnosed with it.” (Medical Officer)

### Precipitated by suppressed immunity.

Health workers narrated that both pneumonia and malnutrition worsened under suppressed immunity. Many of the children whose immunity is weak are more prone to having at least one of these conditions, and the presence of any of these conditions in a child leads to the other.

“Most of the children with acute severe malnutrition have pneumonia because of the low immunity and the GI flora most of them that are non-pathogenic some are aspirated as respiratory flora which are non-pathogenic and once, they get all these they are prone to getting severe pneumonia.” (Senior Housing Officer)

“Anyone who has managed malnutrition will know that it causes immune suppression, and it also causes you to get all those conditions. Most of the colleagues that I have been with have shown that they know how to manage, and the other thing is that when we were still medical students, they used to ask us questions to see whether we understand. In addition, I think they know the relationship between malnutrition and pneumonia”. (Medical Officer)

#### Difficulty in breathing and reduced food intake

Health workers also emphasized the effect of pneumonia causing difficulty in breathing. They were keen to comment on the fact that regardless of recurrence of pneumonia, children find difficulty in breathing with any pneumonia attack, and this affects food intake.

“Those with pneumonia even if it’s not recurring, they usually have difficulty in breathing, so their intake of food is also reduced. When a child is with severe pneumonia during that time, they can’t eat for various reasons, and they can’t even have the energy to feed. Even when you try to feed them, they are at risk of aspiration, and this happens often with severe pneumonia. If they take long in that stage, they can develop malnutrition”(Nurse).

Some participants in the study noted that in some circumstance pneumonia increases the risk of malnutrition. The participants believed that when a child suffers pneumonia, their immunity is weakened, and this leads to chances of becoming malnourished. The health workers strive to keep feeding the child when ill to ensure they have an adequate intake of feeds.

“Many of my colleagues believe that pneumonia affects nutrition that’s why when a child comes in with pneumonia, we labour to attend to it that’s the child’s intake is adequate, we labour to adequately prevent the child from getting malnourished. Yeah, staff know that pneumonia increases the risk of getting malnutrition more so if a child keeps on getting recurrent pneumonia.” (Senior Housing Officer)

#### Use of Protocols and Guidelines

In relation to practices, most health workers follow existing local and global clinical guidelines or protocols in managing severe malnutrition and severe pneumonia in children.

“Yes, there’s treatment protocol for severe pneumonia or from the classification for example cough, fever, the distress or what I mentioned earlier. We followed the WHO and the Uganda clinical guidelines for severe pneumonia treatment.” (Medical officer)

In relation to attitude, apathy was highlighted as a major attitude mothers exhibit towards their children. Poor attitudes towards seeking professional care and following health workers’ guidance delays access to healthcare and contributes to the severity of disease. Some caregiver attitudes were linked to prioritizing the treatment of pneumonia over feeding and growth)

“There’s a very big link between malnutrition and pneumonia because of the attitude of the mothers from home not even in the hospital because by the time the mother is coming to the hospital, the child is already malnourished.” (Nurse)

Failure to follow health worker guidelines by the caregivers and the negative attitude towards conventional feeding practices like breast feeding affects the child’s health status making them prone to pneumonia and malnutrition.

“Even the mothers don’t believe or understand even when you tell them to breast feed the child or feed the child on a specific meal, they don’t want. They think that they are starving their children so when they abandon all that it makes the children malnourished. When these mothers treat their children at the clinic they mind about treatment and don’t mind assessing the child’s weight so for them they are after getting the treatment and the child becomes better but, in the end, the child will become malnourished.”(Nurse)

## Discussion

This study assessed attitudes, practices and understanding of caregivers and health workers regarding the relationship between severe pneumonia and malnutrition. We found that caregivers had some understanding of the link between malnutrition and severe pneumonia. While some thought malnutrition increased the risk of pneumonia, and pneumonia increased the risk of pneumonia by reducing children’s appetite and intake, they also thought a balanced diet and breast feeding could protect children from both illnesses. Some caregivers however thought there was no link between the two conditions, while others expressed that they didn’t know anything about the relationship between the two conditions. Health workers expressed a good understanding of the relationship between the two conditions and expressed that they felt some caregivers didn’t truly take the conditions seriously and seek care in time.

The finding that some caregivers not only expressed a good understanding of the link between severe pneumonia and malnutrition but also took deliberate steps to reduce the risk of malnutrition in their children through improving diet and breastfeeding more frequently was interesting. Unfortunately, this might not necessarily reflect what caregivers do to support their children away from the watchful eyes of the health workers, since they often quickly to return to work to provide for their families. Some caregivers confessed that they didn’t know anything about both conditions as their children had only been hospitalized for the first time, while others expressed that many caregivers, they know don’t take their sick children to hospital. This is not surprising considering that even in other sub-Saharan African countries where even adequate knowledge of severe pneumonia symptoms was displayed, health seeking behaviour was as low as 30% (13). The health workers felt that caregivers did not fully understand that their children were at increased risk of developing severe malnutrition when they had pneumonia and vice versa, so they delayed seeking medical attention, or even worse, sought alternative forms of care altogether, a common finding in developing countries (14). Inadequate knowledge on the other hand is also known to contribute to childhood mortality (15). Caregivers who seek care elsewhere also miss the opportunity to interact with health workers to get useful information on the conditions, and on breaking the cycle, which unfortunately significantly increases the risk of childhood mortality (16, 17). Infant and young child feeding education at all interaction points with health workers could be fill gaps in caregiver knowledge and practices. Gaps in caregiver knowledge and practices on general principles of infant and young child feeding increases the likelihood of poor attitudes and practices in sick children (18).

Health workers of all the cadres interviewed in this study expressed a good understanding of the fatal relationship between severe pneumonia and malnutrition and the mechanisms or pathways involved. However, this did not seem to affect their practices as they did not point out ways in which this changed the way they managed patients inflicted by either condition to prevent the risk of progression. For example, knowing that a patient with severe pneumonia is at risk of severe malnutrition should ideally increase the index of suspension for developing, prompting the health worker to screen for malnutrition at admission and discharge, and increase feeding/nutritional counselling specific for caregivers of such patients. However, as with other sick children, health workers barely assess or specifically counsel caregivers on feeding (10). Chaturvedi et al. also found that knowledge on malnutrition among health workers did not necessarily translate into practice (19). Furthermore, the knowledge of some cadres of health workers may be less than adequate and affect their attitudes and practices, such as meticulous screening of children with severe pneumonia for malnutrition and targeted counselling. Similar results were found among nurses in Ghana who felt nutritional assessment was not their responsibility even if they knew when and how to do it (20). On the other hand, even health workers who counsel caregivers on feeding practices might not be specific enough on special feeding requirements for children with severe pneumonia, as the guidelines are not specific enough regarding the care of children with severe pneumonia at risk of malnutrition (9).

This study explored understanding, attitudes, and practices towards the fatal relationship between severe pneumonia and malnutrition, however, it is not without limitations. First, were unable to access all the different cadres of health workers involved in the management of the children given the staffing of the national referral hospital. However, we covered the key cadres including nurses, medical officers, and paediatric residents. Second, researcher bias could have influenced the findings of the study. However, we used pre-tested, structured guides for the FDGs and KIIs, and had a note-taker present as well. Focus group discussions also presented a challenge given the COVID-19 pandemic, however all the necessary precaution including use of face masks and social distancing were taken to solicit as much information as possible while keeping respondents involved safe.

## Conclusion

Both caregivers and health workers were aware of the relationship between severe pneumonia and malnutrition to varying degrees. Significant gaps in understanding of the deadly relationship between severe pneumonia and malnutrition still exist among caregivers and this calls for more health education and mass sensitization. Clarifying practice guidelines could further enhance attitudes and practices of health workers which are crucial in the fight against preventable pneumonia deaths among children.

## Data Availability

The anonymized codebooks used during the analysis for the current study are available from the corresponding author on reasonable request.
